# The triclinic form of di-μ-aqua-bis­[diaqua­bis­(thio­cyanato-κ*N*)iron(II)]–1,4-bis­(4*H*-1,2,4-triazol-4-yl)benzene (1/3)

**DOI:** 10.1107/S1600536812026141

**Published:** 2012-07-10

**Authors:** Pan Yang, Bin Ding, Gui-Xiang Du

**Affiliations:** a Tianjin Key Laboratory of Structure and Performance for Functional Molecule, Tianjin Normal University, Tianjin 300071, People’s Republic of China

## Abstract

In the title compound, [Fe_2_(NCS)_4_(H_2_O)_6_]·3C_10_H_8_N_6_, the centrosymmetric dinuclear complex contains two Fe^II^ ions bridged by two aqua ligand O atoms, forming a four-membered ring. The slightly distorted octa­hedral coordination environment of the two Fe^II^ ions is completed by two monodentate aqua ligands and two thio­cyanate ligands. One of the 1,4-bis­(4*H*-1,2,4-triazol-4-yl)benzene mol­ecules lies across an inversion center. In the crystal, O—H⋯N hydrogen bonds connect the components, forming a two-dimensional network parallel to (011). In addition, π–π stacking inter­actions involving the benzene and triazole rings, with centroid–centroid distances in the range 3.502 (5)—3.787 (6) Å, connect the two-dimensional hydrogen-bonded network into a three-dimensional network.

## Related literature

For details of compounds containing a diiron center, see: Hsu *et al.* (1999 ▶); Zheng *et al.* (1999 ▶); MacMurdo *et al.* (2000 ▶); Yoon *et al.* (2004 ▶). For related multicompent di­oxy­gen dependent enzymes including toluene mono­oxygenase, see: Sazinsky *et al.* (2004 ▶). For related multicompent di­oxy­gen dependent enzymes including the *R*_2_ subunit of ribonucleotide reductase, see: Nordlund & Eklund (1993 ▶); Stubbe & Van der Donk (1998 ▶). For the monoclinic form of the title compound, see: Liu *et al.* (2012 ▶).
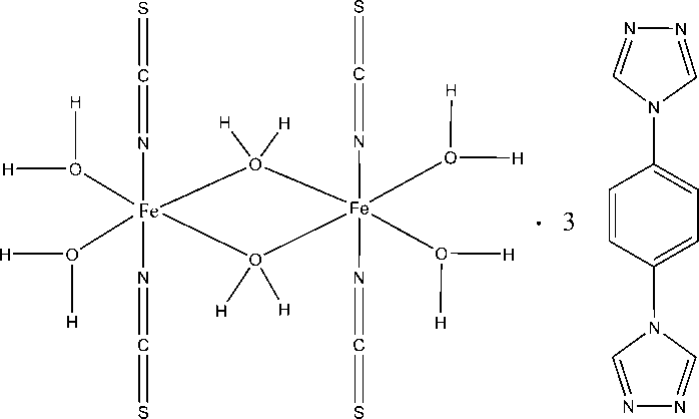


.

## Experimental

### 

#### Crystal data

[Fe_2_(NCS)_4_(H_2_O)_6_]·3C_10_H_8_N_6_
*M* *_r_* = 1088.79
Triclinic, 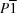

*a* = 7.8335 (6) Å
*b* = 10.9081 (8) Å
*c* = 13.8067 (10) Å
α = 68.999 (1)°
β = 84.952 (1)°
γ = 83.355 (1)°
*V* = 1092.62 (14) Å^3^
*Z* = 1
Mo *K*α radiation
μ = 0.93 mm^−1^
*T* = 173 K
0.18 × 0.14 × 0.13 mm

#### Data collection

Bruker APEXII CCD diffractometer
Absorption correction: multi-scan (*SADABS*; Sheldrick, 1996 ▶) *T*_min_ = 0.851, *T*_max_ = 0.889
5619 measured reflections
3827 independent reflections
3435 reflections with *I* > 2σ(*I*)
*R*_int_ = 0.019

#### Refinement

*R*[*F* ^2^ > 2σ(*F* ^2^)] = 0.031
*wR*(*F* ^2^) = 0.077
*S* = 1.03
3827 reflections
307 parameters
2 restraints
H-atom parameters constrained
Δρ_max_ = 0.30 e Å^−3^
Δρ_min_ = −0.43 e Å^−3^

### 

Data collection: *APEX2* (Bruker, 2007 ▶); cell refinement: *SAINT* (Bruker, 2007 ▶); data reduction: *SAINT*; program(s) used to solve structure: *SHELXS97* (Sheldrick, 2008 ▶); program(s) used to refine structure: *SHELXL97* (Sheldrick, 2008 ▶); molecular graphics: *SHELXTL* (Sheldrick, 2008 ▶) and *DIAMOND* (Brandenburg, 1999 ▶); software used to prepare material for publication: *publCIF* (Westrip, 2010 ▶).

## Supplementary Material

Crystal structure: contains datablock(s) global, I. DOI: 10.1107/S1600536812026141/lh5483sup1.cif

Structure factors: contains datablock(s) I. DOI: 10.1107/S1600536812026141/lh5483Isup2.hkl

Additional supplementary materials:  crystallographic information; 3D view; checkCIF report

## Figures and Tables

**Table 1:** Selected bond lengths (Å)

Fe1—N10	2.0865 (18)
Fe1—N11	2.0968 (18)
Fe1—O3	2.1011 (15)
Fe1—O1	2.1097 (14)
Fe1—O2^i^	2.2552 (14)
Fe1—O2	2.2748 (15)

Symmetry code: (i) 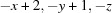.

**Table 2:** Hydrogen-bond geometry (Å, °)

*D*—H⋯*A*	*D*—H	H⋯*A*	*D*⋯*A*	*D*—H⋯*A*
O1—H1*A*⋯N3^ii^	0.84	1.94	2.784 (2)	177
O1—H1*B*⋯N6^iii^	0.84	1.94	2.774 (2)	175
O2—H2*A*⋯N8^i^	0.99	1.86	2.838 (2)	168
O2—H2*B*⋯N9^ii^	0.99	1.85	2.824 (2)	168
O3—H3*A*⋯N5^iv^	0.84	2.01	2.843 (2)	174
O3—H3*B*⋯N2	0.84	2.00	2.834 (2)	174

Symmetry codes: (i) 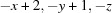; (ii) 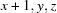; (iii) 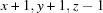; (iv) 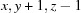.

**Fig. 1.**
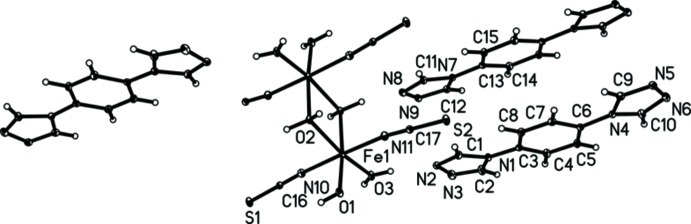
The molecular structure of the title compound, showing 30% probability displacement ellipsoids. Unlabeled atoms in the dinuclear complex and one of the 1,4-Bis(4H-1,2,4-triazol-4-yl)benzene tri-solvate molecules are related by the symmetry codes (-x+2, -y+1, -z) and (-x+1, -y, -z+1), respectively.

**Fig. 2.**
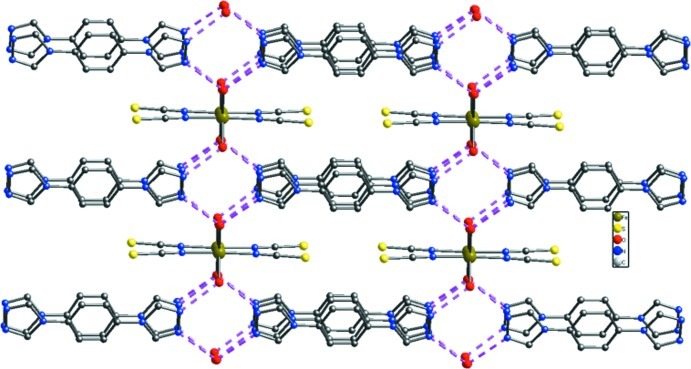
The two-dimensional layered structure of the title complex. Purple Dashed lines indicate donor acceptor distances of the hydrogen bonds. H atoms are not shown.

## supplementary crystallographic information

### Comment

The diiron unit, with a carboxylate-rich coordination environment, continues to attract considerable attention due to the enzyme catalysis activity, which occurs in related multicompent dioxygen dependent enzymes, including toluene monooxy-genase (Sazinsky *et al.*, 2004) and the R2 subunit of ribonucleotide reductase (Stubbe & Van der Donk, 1998; Nordlund & Eklund, 1993). With the development of compounds that contain the diiron center, the structure of a series of Fe2(II,II) (MacMurdo *et al.*, 2000), Fe2(III,III) (Zheng *et al.*, 1999) and Fe2(III,IV) (Hsu *et al.*, 1999) complexes with a central Fe_2_O_2_ four-membered ring have been obtained. Compared to the chelating to iron atoms with carboxylic oxygen atoms, it is rarely reported that the four-membered center includes both aqua oxygen atoms. In order to explore further details of the coordinated environment of the diiron system, the title complex was synthesized and its crystal structure is presented herein.

The molecular structure of the title complex is shown in Fig. 1. The dinuclear complex structure comprises two Fe^II^ ions related by a crystallographic inversion center and bridged by two aqua oxygen atoms to form a four-membered core. Both Fe^II^ ions are in a slightly distorted octahedral coordination environment. The separation between the Fe^II^ ions is 3.487 (1) Å, compared to 3.0430 (7) Å reported previously (Yoon *et al.*, 2004) possibly owing to the absence of the carboxylate ligands in the title compound. Moreover, the Fe···Fe distance is comparatively different from that of diiron compounds containing higher valences of iron (MacMurdo *et al.*, 2000; Zheng *et al.*, 1999; Hsu *et al.*, 1999). In the crystal, O—H···N hydrogen bonds connect the components of the structure to form a two-dimensional network parallel to (011) (see Fig. 2). In addition, π···π stacking interactions involving the benzene and triazole rings with centroid to centroid distances in the range 3.502 (5)—3.787 (6) Å connect the two-dimensional hydrogen-bonded network into a three-dimensional network.

### Experimental

The compound was synthesized under hydrothermal conditions. A mixture of *L* (*L* = 1,4-Bis(4*H*-1,2,4-triazol-4-yl)benzene) (0.3 mmol, 0.0636 g), FeSO_4_.7H_2_O (0.1 mmol, 0.028 g), KSCN (0.2 mmol, 0.019 g) and water (10 ml) was placed in a 25 ml acid digestion bomb and heated at 393 K for two days, then cooled to room temperature over three days. After being washed by 5 ml water twice, colorless block-shaped crystals of the title compound were obtained.

### Refinement

The water H atoms were located in a Fourier difference map and refined subject to an O—H restraint 0.88 (1) Å and an H···H restraint of 1.42 (2) Å. Other H atoms were allowed to ride on their parent atoms with C—H distances of 0.93 Å (*U*_iso_(H) = 1.2Ueq(C)).

### Crystal data

**Table:**

[Fe_2_(NCS)_4_(H_2_O)_6_]·3C_10_H_8_N_6_	*Z* = 1
*M**_r_* = 1088.79	*F*(000) = 558
Triclinic, *P*1	*D*_x_ = 1.655 Mg m^−^^3^
Hall symbol: -P 1	Mo *K*α radiation, λ = 0.71073 Å
*a* = 7.8335 (6) Å	Cell parameters from 2875 reflections
*b* = 10.9081 (8) Å	θ = 3.0–28.4°
*c* = 13.8067 (10) Å	µ = 0.93 mm^−^^1^
α = 68.999 (1)°	*T* = 173 K
β = 84.952 (1)°	Block, colourless
γ = 83.355 (1)°	0.18 × 0.14 × 0.13 mm
*V* = 1092.62 (14) Å^3^	

### Data collection

**Table:**

Bruker APEXII CCD diffractometer	3827 independent reflections
Radiation source: fine-focus sealed tube	3435 reflections with *I* > 2σ(*I*)
Graphite monochromator	*R*_int_ = 0.019
φ and ω scans	θ_max_ = 25.0°, θ_min_ = 1.6°
Absorption correction: multi-scan (*SADABS*; Sheldrick, 1996)	*h* = −8→9
*T*_min_ = 0.851, *T*_max_ = 0.889	*k* = −9→12
5619 measured reflections	*l* = −15→16

### Refinement

**Table:**

Refinement on *F*^2^	2 restraints
Least-squares matrix: full	H-atom parameters constrained
*R*[*F*^2^ > 2σ(*F*^2^)] = 0.031	*w* = 1/[σ^2^(*F*_o_^2^) + (0.0345*P*)^2^ + 0.7404*P*] where *P* = (*F*_o_^2^ + 2*F*_c_^2^)/3
*wR*(*F*^2^) = 0.077	(Δ/σ)_max_ = 0.001
*S* = 1.03	Δρ_max_ = 0.30 e Å^−^^3^
3827 reflections	Δρ_min_ = −0.43 e Å^−^^3^
307 parameters	

### Special details

**Table:**

Geometry. All e.s.d.'s (except the e.s.d. in the dihedral angle between two l.s. planes) are estimated using the full covariance matrix. The cell e.s.d.'s are taken into account individually in the estimation of e.s.d.'s in distances, angles and torsion angles; correlations between e.s.d.'s in cell parameters are only used when they are defined by crystal symmetry. An approximate (isotropic) treatment of cell e.s.d.'s is used for estimating e.s.d.'s involving l.s. planes.
Refinement. Refinement of *F*^2^ against ALL reflections. The weighted *R*-factor *wR* and goodness of fit *S* are based on *F*^2^, conventional *R*-factors *R* are based on *F*, with *F* set to zero for negative *F*^2^. The threshold expression of *F*^2^ > σ(*F*^2^) is used only for calculating *R*-factors(gt) *etc*. and is not relevant to the choice of reflections for refinement. *R*-factors based on *F*^2^ are statistically about twice as large as those based on *F*, and *R*- factors based on ALL data will be even larger.

### Fractional atomic coordinates and isotropic or equivalent isotropic displacement parameters (Å^2^)

**Table:**

	*x*	*y*	*z*	*U*_iso_*/*U*_eq_	
Fe1	0.93995 (4)	0.61553 (3)	0.06003 (2)	0.01245 (10)	
S1	0.94951 (7)	0.98407 (6)	−0.27085 (4)	0.01874 (14)	
S2	0.94707 (7)	0.29730 (6)	0.41566 (4)	0.01899 (14)	
O1	1.08753 (19)	0.72399 (15)	0.11568 (11)	0.0168 (3)	
H1A	1.1503	0.6859	0.1662	0.025*	
H1B	1.1432	0.7829	0.0721	0.025*	
O2	1.17972 (18)	0.51345 (14)	0.00652 (10)	0.0135 (3)	
H2A	1.2497	0.5774	−0.0469	0.020*	
H2B	1.2517	0.4605	0.0653	0.020*	
O3	0.68912 (19)	0.68356 (15)	0.09608 (11)	0.0172 (3)	
H3A	0.6264	0.7424	0.0535	0.026*	
H3B	0.6237	0.6411	0.1452	0.026*	
N1	0.3803 (2)	0.43716 (17)	0.42713 (13)	0.0149 (4)	
N2	0.4701 (2)	0.55412 (18)	0.26843 (13)	0.0166 (4)	
N3	0.2969 (2)	0.58853 (19)	0.28071 (14)	0.0212 (4)	
N4	0.3748 (2)	0.05660 (17)	0.83062 (13)	0.0133 (4)	
N5	0.4598 (2)	−0.11802 (18)	0.96406 (13)	0.0165 (4)	
N6	0.2857 (2)	−0.08517 (18)	0.97992 (14)	0.0188 (4)	
N7	0.5005 (2)	0.19396 (17)	0.29899 (13)	0.0116 (4)	
N8	0.5907 (2)	0.33244 (18)	0.14775 (13)	0.0151 (4)	
N9	0.4119 (2)	0.34901 (17)	0.15464 (13)	0.0150 (4)	
N10	0.9418 (2)	0.76782 (18)	−0.08416 (14)	0.0164 (4)	
N11	0.9443 (2)	0.46757 (18)	0.20753 (14)	0.0168 (4)	
C1	0.5168 (3)	0.4650 (2)	0.35647 (16)	0.0172 (5)	
H1	0.6307	0.4247	0.3696	0.021*	
C2	0.2474 (3)	0.5182 (2)	0.37532 (17)	0.0207 (5)	
H2	0.1332	0.5231	0.4041	0.025*	
C3	0.3786 (3)	0.3430 (2)	0.53126 (16)	0.0138 (4)	
C4	0.2299 (3)	0.3324 (2)	0.59539 (16)	0.0173 (5)	
H4	0.1291	0.3888	0.5708	0.021*	
C5	0.2281 (3)	0.2398 (2)	0.69513 (16)	0.0159 (5)	
H5	0.1264	0.2325	0.7390	0.019*	
C6	0.3758 (3)	0.1580 (2)	0.73034 (15)	0.0135 (4)	
C7	0.5257 (3)	0.1709 (2)	0.66666 (16)	0.0160 (5)	
H7	0.6275	0.1161	0.6917	0.019*	
C8	0.5268 (3)	0.2630 (2)	0.56752 (16)	0.0159 (5)	
H8	0.6291	0.2717	0.5241	0.019*	
C9	0.5101 (3)	−0.0323 (2)	0.87559 (16)	0.0167 (5)	
H9	0.6244	−0.0314	0.8462	0.020*	
C10	0.2392 (3)	0.0185 (2)	0.89994 (16)	0.0173 (5)	
H10	0.1260	0.0614	0.8913	0.021*	
C11	0.6395 (3)	0.2405 (2)	0.23378 (16)	0.0146 (5)	
H11	0.7560	0.2098	0.2493	0.017*	
C12	0.3619 (3)	0.2663 (2)	0.24462 (16)	0.0144 (5)	
H12	0.2452	0.2573	0.2692	0.017*	
C13	0.5004 (3)	0.0950 (2)	0.40112 (15)	0.0119 (4)	
C14	0.3452 (3)	0.0654 (2)	0.45848 (16)	0.0147 (5)	
H14	0.2398	0.1101	0.4297	0.018*	
C15	0.6553 (3)	0.0293 (2)	0.44258 (16)	0.0147 (5)	
H15	0.7608	0.0493	0.4032	0.018*	
C16	0.9452 (3)	0.8577 (2)	−0.16137 (16)	0.0128 (4)	
C17	0.9453 (3)	0.3973 (2)	0.29352 (16)	0.0139 (4)	

### Atomic displacement parameters (Å^2^)

**Table:**

	*U*^11^	*U*^22^	*U*^33^	*U*^12^	*U*^13^	*U*^23^
Fe1	0.01271 (17)	0.01269 (17)	0.01021 (16)	−0.00163 (13)	−0.00107 (12)	−0.00164 (13)
S1	0.0169 (3)	0.0181 (3)	0.0141 (3)	−0.0007 (2)	−0.0003 (2)	0.0026 (2)
S2	0.0171 (3)	0.0201 (3)	0.0129 (3)	0.0002 (2)	−0.0008 (2)	0.0019 (2)
O1	0.0173 (8)	0.0179 (8)	0.0124 (7)	−0.0047 (6)	−0.0022 (6)	−0.0008 (6)
O2	0.0135 (7)	0.0140 (8)	0.0103 (7)	−0.0012 (6)	−0.0013 (6)	−0.0008 (6)
O3	0.0154 (8)	0.0180 (8)	0.0118 (7)	0.0000 (6)	0.0011 (6)	0.0017 (6)
N1	0.0184 (10)	0.0130 (9)	0.0108 (9)	−0.0002 (8)	−0.0013 (8)	−0.0015 (7)
N2	0.0207 (10)	0.0147 (10)	0.0144 (9)	−0.0022 (8)	−0.0001 (8)	−0.0050 (8)
N3	0.0197 (10)	0.0221 (11)	0.0194 (10)	−0.0035 (8)	−0.0049 (8)	−0.0029 (8)
N4	0.0148 (9)	0.0123 (9)	0.0118 (8)	−0.0008 (8)	−0.0006 (7)	−0.0033 (7)
N5	0.0194 (10)	0.0140 (9)	0.0153 (9)	0.0001 (8)	−0.0028 (8)	−0.0041 (8)
N6	0.0207 (10)	0.0175 (10)	0.0161 (9)	−0.0044 (8)	−0.0018 (8)	−0.0024 (8)
N7	0.0128 (9)	0.0110 (9)	0.0100 (8)	−0.0010 (7)	−0.0014 (7)	−0.0023 (7)
N8	0.0150 (9)	0.0140 (9)	0.0149 (9)	−0.0017 (8)	−0.0024 (7)	−0.0028 (8)
N9	0.0164 (10)	0.0130 (9)	0.0139 (9)	−0.0017 (8)	−0.0032 (7)	−0.0022 (8)
N10	0.0132 (10)	0.0173 (10)	0.0171 (9)	0.0003 (8)	−0.0016 (8)	−0.0042 (8)
N11	0.0143 (10)	0.0187 (10)	0.0162 (9)	−0.0023 (8)	−0.0004 (8)	−0.0043 (8)
C1	0.0179 (12)	0.0184 (12)	0.0156 (11)	−0.0025 (9)	0.0015 (9)	−0.0066 (9)
C2	0.0167 (12)	0.0229 (13)	0.0179 (11)	−0.0020 (10)	−0.0011 (10)	−0.0014 (10)
C3	0.0172 (11)	0.0125 (11)	0.0131 (10)	−0.0031 (9)	−0.0010 (9)	−0.0055 (9)
C4	0.0140 (11)	0.0176 (12)	0.0161 (11)	0.0034 (9)	−0.0016 (9)	−0.0020 (9)
C5	0.0121 (11)	0.0179 (12)	0.0150 (10)	−0.0004 (9)	0.0025 (9)	−0.0034 (9)
C6	0.0178 (11)	0.0121 (11)	0.0119 (10)	−0.0026 (9)	−0.0019 (9)	−0.0049 (9)
C7	0.0151 (11)	0.0154 (11)	0.0165 (11)	0.0022 (9)	−0.0026 (9)	−0.0050 (9)
C8	0.0135 (11)	0.0171 (11)	0.0159 (11)	−0.0011 (9)	0.0010 (9)	−0.0050 (9)
C9	0.0182 (12)	0.0160 (11)	0.0156 (11)	0.0008 (9)	−0.0043 (9)	−0.0051 (9)
C10	0.0154 (12)	0.0182 (12)	0.0161 (11)	−0.0025 (9)	−0.0014 (9)	−0.0029 (9)
C11	0.0134 (11)	0.0164 (11)	0.0130 (10)	−0.0021 (9)	0.0000 (9)	−0.0040 (9)
C12	0.0149 (11)	0.0144 (11)	0.0130 (10)	0.0001 (9)	−0.0030 (9)	−0.0037 (9)
C13	0.0151 (11)	0.0104 (10)	0.0105 (10)	−0.0022 (9)	−0.0026 (8)	−0.0032 (8)
C14	0.0114 (11)	0.0158 (11)	0.0153 (10)	0.0000 (9)	−0.0023 (9)	−0.0036 (9)
C15	0.0125 (11)	0.0165 (11)	0.0137 (10)	−0.0018 (9)	0.0014 (9)	−0.0039 (9)
C16	0.0104 (10)	0.0146 (10)	0.0131 (9)	0.0001 (9)	−0.0005 (8)	−0.0048 (7)
C17	0.0121 (11)	0.0137 (11)	0.0148 (9)	−0.0002 (9)	0.0003 (9)	−0.0041 (8)

### Geometric parameters (Å, º)

**Table:**

Fe1—N10	2.0865 (18)	N7—C13	1.436 (3)
Fe1—N11	2.0968 (18)	N8—C11	1.304 (3)
Fe1—O3	2.1011 (15)	N8—N9	1.391 (3)
Fe1—O1	2.1097 (14)	N9—C12	1.305 (3)
Fe1—O2^i^	2.2552 (14)	N10—C16	1.162 (3)
Fe1—O2	2.2748 (15)	N11—C17	1.160 (3)
S1—C16	1.641 (2)	C1—H1	0.9500
S2—C17	1.648 (2)	C2—H2	0.9500
O1—H1A	0.8401	C3—C8	1.384 (3)
O1—H1B	0.8401	C3—C4	1.389 (3)
O2—Fe1^i^	2.2552 (14)	C4—C5	1.385 (3)
O2—H2A	0.9900	C4—H4	0.9500
O2—H2B	0.9900	C5—C6	1.386 (3)
O3—H3A	0.8401	C5—H5	0.9500
O3—H3B	0.8401	C6—C7	1.393 (3)
N1—C2	1.357 (3)	C7—C8	1.377 (3)
N1—C1	1.366 (3)	C7—H7	0.9500
N1—C3	1.437 (3)	C8—H8	0.9500
N2—C1	1.306 (3)	C9—H9	0.9500
N2—N3	1.377 (3)	C10—H10	0.9500
N3—C2	1.306 (3)	C11—H11	0.9500
N4—C10	1.362 (3)	C12—H12	0.9500
N4—C9	1.377 (3)	C13—C15	1.393 (3)
N4—C6	1.430 (3)	C13—C14	1.396 (3)
N5—C9	1.305 (3)	C14—C15^ii^	1.386 (3)
N5—N6	1.388 (3)	C14—H14	0.9500
N6—C10	1.308 (3)	C15—C14^ii^	1.386 (3)
N7—C11	1.372 (3)	C15—H15	0.9500
N7—C12	1.373 (3)		
			
N10—Fe1—N11	177.40 (7)	N1—C1—H1	124.5
N10—Fe1—O3	90.67 (6)	N3—C2—N1	111.2 (2)
N11—Fe1—O3	89.72 (6)	N3—C2—H2	124.4
N10—Fe1—O1	88.84 (6)	N1—C2—H2	124.4
N11—Fe1—O1	88.55 (6)	C8—C3—C4	120.1 (2)
O3—Fe1—O1	101.03 (6)	C8—C3—N1	119.39 (19)
N10—Fe1—O2^i^	91.03 (6)	C4—C3—N1	120.48 (19)
N11—Fe1—O2^i^	91.56 (6)	C5—C4—C3	120.2 (2)
O3—Fe1—O2^i^	87.55 (6)	C5—C4—H4	119.9
O1—Fe1—O2^i^	171.43 (6)	C3—C4—H4	119.9
N10—Fe1—O2	89.61 (6)	C4—C5—C6	119.4 (2)
N11—Fe1—O2	90.60 (6)	C4—C5—H5	120.3
O3—Fe1—O2	166.90 (5)	C6—C5—H5	120.3
O1—Fe1—O2	92.07 (6)	C5—C6—C7	120.17 (19)
O2^i^—Fe1—O2	79.35 (5)	C5—C6—N4	120.60 (19)
Fe1—O1—H1A	120.8	C7—C6—N4	119.19 (19)
Fe1—O1—H1B	118.2	C8—C7—C6	120.2 (2)
H1A—O1—H1B	106.9	C8—C7—H7	119.9
Fe1^i^—O2—Fe1	100.65 (5)	C6—C7—H7	119.9
Fe1^i^—O2—H2A	111.6	C7—C8—C3	119.8 (2)
Fe1—O2—H2A	111.6	C7—C8—H8	120.1
Fe1^i^—O2—H2B	111.6	C3—C8—H8	120.1
Fe1—O2—H2B	111.6	N5—C9—N4	110.8 (2)
H2A—O2—H2B	109.4	N5—C9—H9	124.6
Fe1—O3—H3A	124.5	N4—C9—H9	124.6
Fe1—O3—H3B	125.3	N6—C10—N4	111.0 (2)
H3A—O3—H3B	106.9	N6—C10—H10	124.5
C2—N1—C1	103.71 (18)	N4—C10—H10	124.5
C2—N1—C3	128.44 (19)	N8—C11—N7	111.06 (19)
C1—N1—C3	127.84 (18)	N8—C11—H11	124.5
C1—N2—N3	106.87 (18)	N7—C11—H11	124.5
C2—N3—N2	107.16 (18)	N9—C12—N7	110.95 (19)
C10—N4—C9	104.00 (18)	N9—C12—H12	124.5
C10—N4—C6	128.60 (18)	N7—C12—H12	124.5
C9—N4—C6	127.22 (18)	C15—C13—C14	120.34 (19)
C9—N5—N6	107.03 (17)	C15—C13—N7	119.82 (19)
C10—N6—N5	107.25 (18)	C14—C13—N7	119.84 (18)
C11—N7—C12	103.73 (17)	C15^ii^—C14—C13	120.01 (19)
C11—N7—C13	128.06 (17)	C15^ii^—C14—H14	120.0
C12—N7—C13	128.19 (18)	C13—C14—H14	120.0
C11—N8—N9	107.13 (17)	C14^ii^—C15—C13	119.7 (2)
C12—N9—N8	107.12 (17)	C14^ii^—C15—H15	120.2
C16—N10—Fe1	175.78 (17)	C13—C15—H15	120.2
C17—N11—Fe1	172.21 (17)	N10—C16—S1	179.6 (2)
N2—C1—N1	111.1 (2)	N11—C17—S2	179.9 (3)
N2—C1—H1	124.5		
			
N10—Fe1—O2—Fe1^i^	−91.12 (6)	C4—C5—C6—N4	−176.34 (18)
N11—Fe1—O2—Fe1^i^	91.48 (6)	C10—N4—C6—C5	5.2 (3)
O3—Fe1—O2—Fe1^i^	0.2 (3)	C9—N4—C6—C5	179.4 (2)
O1—Fe1—O2—Fe1^i^	−179.95 (5)	C10—N4—C6—C7	−172.6 (2)
O2^i^—Fe1—O2—Fe1^i^	0.0	C9—N4—C6—C7	1.7 (3)
C1—N2—N3—C2	−0.2 (2)	C5—C6—C7—C8	−1.5 (3)
C9—N5—N6—C10	0.3 (2)	N4—C6—C7—C8	176.30 (18)
C11—N8—N9—C12	0.1 (2)	C6—C7—C8—C3	0.1 (3)
N11—Fe1—N10—C16	−18 (3)	C4—C3—C8—C7	1.4 (3)
O3—Fe1—N10—C16	80 (2)	N1—C3—C8—C7	−178.93 (18)
O1—Fe1—N10—C16	−21 (2)	N6—N5—C9—N4	−0.7 (2)
O2^i^—Fe1—N10—C16	168 (2)	C10—N4—C9—N5	0.8 (2)
O2—Fe1—N10—C16	−113 (2)	C6—N4—C9—N5	−174.65 (18)
N10—Fe1—N11—C17	34 (2)	N5—N6—C10—N4	0.2 (2)
O3—Fe1—N11—C17	−64.2 (12)	C9—N4—C10—N6	−0.6 (2)
O1—Fe1—N11—C17	36.9 (12)	C6—N4—C10—N6	174.75 (18)
O2^i^—Fe1—N11—C17	−151.7 (12)	N9—N8—C11—N7	0.0 (2)
O2—Fe1—N11—C17	128.9 (12)	C12—N7—C11—N8	0.0 (2)
N3—N2—C1—N1	0.7 (2)	C13—N7—C11—N8	−178.74 (18)
C2—N1—C1—N2	−0.9 (2)	N8—N9—C12—N7	−0.1 (2)
C3—N1—C1—N2	178.46 (18)	C11—N7—C12—N9	0.1 (2)
N2—N3—C2—N1	−0.3 (3)	C13—N7—C12—N9	178.79 (18)
C1—N1—C2—N3	0.7 (2)	C11—N7—C13—C15	−3.3 (3)
C3—N1—C2—N3	−178.62 (19)	C12—N7—C13—C15	178.31 (19)
C2—N1—C3—C8	175.7 (2)	C11—N7—C13—C14	176.49 (19)
C1—N1—C3—C8	−3.5 (3)	C12—N7—C13—C14	−1.9 (3)
C2—N1—C3—C4	−4.6 (3)	C15—C13—C14—C15^ii^	0.3 (3)
C1—N1—C3—C4	176.2 (2)	N7—C13—C14—C15^ii^	−179.45 (18)
C8—C3—C4—C5	−1.4 (3)	C14—C13—C15—C14^ii^	−0.3 (3)
N1—C3—C4—C5	178.86 (19)	N7—C13—C15—C14^ii^	179.45 (18)
C3—C4—C5—C6	0.0 (3)	Fe1—N10—C16—S1	−174 (100)
C4—C5—C6—C7	1.4 (3)	Fe1—N11—C17—S2	−123 (100)

Symmetry codes: (i) −*x*+2, −*y*+1, −*z*; (ii) −*x*+1, −*y*, −*z*+1.

### Hydrogen-bond geometry (Å, º)

**Table:**

*D*—H···*A*	*D*—H	H···*A*	*D*···*A*	*D*—H···*A*
O1—H1*A*···N3^iii^	0.84	1.94	2.784 (2)	177
O1—H1*B*···N6^iv^	0.84	1.94	2.774 (2)	175
O2—H2*A*···N8^i^	0.99	1.86	2.838 (2)	168
O2—H2*B*···N9^iii^	0.99	1.85	2.824 (2)	168
O3—H3*A*···N5^v^	0.84	2.01	2.843 (2)	174
O3—H3*B*···N2	0.84	2.00	2.834 (2)	174

Symmetry codes: (i) −*x*+2, −*y*+1, −*z*; (iii) *x*+1, *y*, *z*; (iv) *x*+1, *y*+1, *z*−1; (v) *x*, *y*+1, *z*−1.

## References

[bb1] Brandenburg, K. (1999). *DIAMOND* Crystal Impact GbR, Bonn, Germany.

[bb2] Bruker (2007). *APEX2* and *SAINT* Bruker AXS Inc., Madison, Wisconsin, USA.

[bb3] Hsu, H. F., Dong, Y., Shu, L., Young, V. G. Jr & Que, L. Jr (1999). *J. Am. Chem. Soc.* **121**, 5230–5237.

[bb13] Liu, Y.-Y., Yang, P. & Ding, B. (2012). *Acta Cryst.* E**68**, m1036–m1037. 10.1107/S160053681202613X PMC3414108 22904715

[bb4] MacMurdo, V. L., Zheng, H. & Que, L. Jr (2000). *Inorg. Chem.* **39**, 2254–2255. 10.1021/ic991482m 12526481

[bb5] Nordlund, P. & Eklund, H. (1993). *J. Mol. Biol.* **232**, 123–164. 10.1006/jmbi.1993.1374 8331655

[bb6] Sazinsky, M. H., Bard, J., Di Donato, A. & Lippard, S. J. (2004). *J. Biol. Chem.* **279**, 30600–30610. 10.1074/jbc.M400710200 15096510

[bb7] Sheldrick, G. M. (1996). *SADABS* University of Göttingen, Germany.

[bb8] Sheldrick, G. M. (2008). *Acta Cryst.* A**64**, 112–122. 10.1107/S0108767307043930 18156677

[bb9] Stubbe, J. & Van der Donk, W. A. (1998). *Chem. Rev.* **98**, 705–762. 10.1021/cr9400875 11848913

[bb10] Westrip, S. P. (2010). *J. Appl. Cryst.* **43**, 920–925.

[bb11] Yoon, S., Kelly, A. E. & Lippard, S. J. (2004). *Polyhedron*, **23**, 2805–2812.

[bb12] Zheng, H., Zang, Y., Dong, Y., Young, V. G. Jr & Que, L. Jr (1999). *J. Am. Chem. Soc.* **121**, 2226–2235.

